# Identity, language and communication concerns of subcultures: The case of Antalya Cretans

**DOI:** 10.1371/journal.pone.0314543

**Published:** 2025-01-06

**Authors:** Pınar ŞİMŞEK

**Affiliations:** Faculty of Education, Akdeniz University, Antalya, Turkey; Karamanoglu Mehmetbey University: Karamanoglu Mehmetbey Universitesi, TÜRKIYE

## Abstract

In today’s world, there is almost no homogeneous culture without interaction, and multiculturalism has become the most important phenomenon for all societies. Therefore, this cultural diversity, consisting of differences in culture, language, identity, religion, etc. has also brought along many problems. In the United Nations Educational, Scientific and Cultural Organization’s in “Convention for the Safeguarding of Intangible Cultural Heritage”, it is stated that identifying, documenting and surveying intangible cultural heritage of communities contributes to social harmony and tolerance. This research has been conducted on the identity, language, and communication of the Cretan in Türkiye. The purpose of the study is to contribute to social peace and tolerance by examining the identity, language, and communication perceptions and concerns of the Crete-origin community living in Türkiye as a subculture group, within the context of multiculturalism and intercultural communication. In this direction, preliminary interviews, observations and semi-structured in-depth interviews were made in the fieldwork carried out between May-July 2024 in the of the Cretan immigrants who have migrated and settled in Antalya at the first settlements one of the world cities with intense multiculturalism. In the study, an answer has been sought to the question “What are the identity and linguistic anxiety, harmony and conflict situations in the communication and interaction of Cretans living in Turkey as a subculture with the dominant culture?”. The findings were subjected to thematic and descriptive analysis and interpreted. It is predicted that the findings and results of this research can be used to contribute to studies aimed at preventing identity, linguistic and cultural incompatibility and possible conflicts caused by increasing migration movements.

## Introduction

Throughout history, many people have had to leave their homeland and migrate for various reasons. As a subculture, they have faced many problems such as identity, language and communication in the countries they live in.

### Multiculturalism, dominant culture, and subculture

Due to the fact that there are hundreds of living languages, ethnic communities and different cultures in the world, and a homogeneous structure is almost unseen except for a few states, it has become almost a necessity for countries to recognize different identities and respect cultural diversity. In this study, rather than defining the concepts, the focus is on the effects of the facts that come to life in these concepts on human life. Multiculturalism has been seen as cultural diversity [1] resulting from the existence of two or more groups with beliefs and practices that create a unique sense of collective identity, often linked to racial, ethnic, or linguistic differences, and its effects on social recognition and identity politics [2] have been discussed.

Multiculturalism should be seen as the ability to live together while preserving differences. Multiculturalism is the recognition and acceptance of the beliefs, ethnicity, language, and religious values that define the identity of social groups. In the study [3] examining the effects of culture on society and the dynamics between dominant culture and subcultures, it is emphasized that subcultures cannot be manipulated by the dominant class or evaluated passively.

The perception that different cultures and languages (subcultures) can lead to the degeneration of cultural elements and language of the dominant culture within the country and damage national unity; On the other hand, the steps that can be taken in line with the concerns of subcultures about losing, preserving and transferring their own culture and languages may cause some negativities and even conflicts. Efforts should be made for integration instead of assimilating or eradicating subcultures and languages as a result of poorly managed migration and integration processes. Integration is closely tied to the identity concept of both newcomers and the host community [4]. Identity, cultural, linguistic diversity and difference within a country; Should it be seen as a factor that increases solidarity, cohesion, desire to live together, better understanding and tolerance of each other and enriching human creativity, or should it be seen as a cause of inter-communal conflicts with negativities such as ignoring each other, assimilation, superiority and marginalization of different cultures and languages? Of course, the expectation here; the state, which is the dominant culture, recognizes, respects and tolerates differences. It is the fact that subcultures and identities comply with the laws and rules of the country they live in and belong to. Only in this case can cultural diversity and difference be a factor that increases the desired peace, tranquility, tolerance and human creativity. Because countries may abuse cultural diversity and differences against each other from time to time due to conflict of interest, causing conflicts between the dominant culture and subcultures.

### Language, identity, and belonging

Language can be seen as the code for the genetic characteristics of an identity. “Language helps to build or end relationships; it reminds you of who you are and where you come from” [5]. Language is the identity of a nation, which is the common expression of its way of life and philosophy of life. The main function of language; It establishes natural, emotional and spiritual bonds between people. At the same time, language has an indispensable importance in the formation and transmission of belief, identity and all cultural values. Identity refers to the value system that individuals and nations highlight and refer to when defining themselves. Belonging is the definition or self-definition of individuals through a relationship of identification with a collective. The mentioned connections are fundamental grounds for individuals to acquire identity. The subjective structure of language constitutes the treasure of identity. To put it more explicitly, every kind of emotion and thought that identity requires exists in language in this respect, language is the most valuable treasure in the formation of identity. The official language becoming the dominant language also strengthens the concept of citizenship [6–9]. People who share common values transmit them to future generations through their language.

### Origins of Cretan immigrants in Antalya

The island of Crete, which is the intersection point of great civilizations, is considered a key location in the Mediterranean due to its geographical position and strategic importance. Therefore, it is understood that the island has been a region that powerful states have wanted to control since ancient times. In the historical process, important cultural flows are seen in the region. The region has been successively under the dominion of the Roman Empire, the Arabs, the Venetians, and the Ottomans. Although every state that has gained sovereignty seeks to establish its own culture in the region, on Crete, a rich culture has emerged from the interaction of local culture with different cultures, giving rise to a significant civilization known as the Cretan Civilization that left its mark on the region. Finally, 17. In Crete, which came under Ottoman rule in the second half of the century, special arrangements specific to the island were made by the Ottomans. Under these conditions, it can be stated that there was a relatively calm period for a long time on the island, which gives the appearance of an intertwined social life [10].

Theologian Professor Theodoros I. Riginiotis [11] have been determined that, for the Muslim population of Crete; “I believe, that the Moslem population of Crete under the Ottoman rule was, in overwhelming majority, Islamized Cretans of Greek and more rarely of Venetian origin.”. While the participants of this research are they state that the Cretans who migrated to Turkey are the descendants of the Turks who were settled in Crete from Anatolia by the Ottoman state. Perhaps this statement; After coming to Turkey, because they know little or no Turkish, they do not accept the accusation made by the local Anatolian people with the expression ‘*Yarım gâvur*’ and they say it to prove that they are Turks too (The term ‘*Yarım Gavur*’ is used in the sense of ‘shunned enemy-stranger’ that Turks give to non-Turks and non-Muslims.). Although the majority of Muslims coming from Crete are Turks, also Muslim communities who also speak Greek have migrated and settled in the southern coasts of Anatolia [12]. The findings of this research indicate that both perspectives have valid points. When the island of Crete came under the rule of the Turks, settlements were made on the island from Anatolia, mostly soldiers and civil servants, in accordance with the state policy of the Ottomans. In this case, the source of the Cretan immigrants living in Antalya consists of Turkish soldiers and officials who settled on the island, local women who converted to Islam and married them, and the native population of Greek and Venetian origin who willingly changed their religion to become Muslims.

The Cretans, who came to Anatolia with two waves of migration, were first settled in provinces such as Izmir, Antalya and Mersin on the Mediterranean and Aegean coasts as a result of forced migration between 1899 and 1912. Between 1897 and 1912, nearly 5,000 Cretan immigrants were settled in Antalya. Among the regions where immigrants from Crete to Antalya have settled are Şarampol District, Sultaniye District in Alanya, Selimiye Village in Old Antalya, and Kadriye Village in Çakallık District [12]. According to Çimrin [13], in 1896, 300 people from the island of Crete were settled in the central Şarampol neighborhood of Antalya, in 1898 294 people in two groups were settled in Alanya, Belek and Kadriye, and in 1908 192 people were settled in the Şarampol area. “Since it was an irregular migration event at the beginning, the Porte had to intervene in the migration issue” [14]. The second wave of migration occurred on the basis of the “Turkish-Greek Exchange Convention and Protocol” of January 30, 1923. The migrants who came as a result of the exchange agreement were also settled in coastal areas [15]. The Cretans who came to Turkey were Cretans who accepted Islam among the people of the island during the period 1645–1908 when the island of Crete was under the rule of the Ottoman Empire, and Turks who went to Crete from Anatolia [10]. The locals and Greeks saw the Muslims of the island as Turks, and they adopted the Turkish identity and called themselves Turks. According to the information obtained from the participant narratives and related literature; The Cretans accepted and used Islam as equivalent to, or even the same meaning as, Turkishness. So much so that they did not say to a person who converted to Islam, ‘He became a Muslim’, but used the phrase ‘He became Turkic (Aftos e Turçepse)’ [10]. Although Cretan immigrants are defined as ‘yarım gâvur’(half stranger) by the local Anatolian people this time due to the fact that they know little or no Turkish after they came to Turkey, it is seen that they willingly and espousing accepted and adopted the citizenship of the Republic of Turkey.

### Languages of Cretan immigrants: Turkish, Cretan (Kritika)

From the book “Crete in Every Aspect” by Prof. Dr. Hakkı Bilgehan, who is also a Cretan immigrant, from the work of theologian Professor Theodoros I. Riginiotis, who studied the Cretan language, and from the opinions of the participants were obtained these data:

Although the language spoken by the Cretans who migrated to Turkey is considered a dialect of Greek, it differs significantly. In the words of Bilgehan [16], although the sentence structure of Cretan is the same as Greek, there are many different words (Table 1).

**Table 1:** Examples of words that are different in Greek and Cretan.

Turkish	Greek	Cretan (Kritika)
çocuk	to pedhi	to pedhi–to kopeli
sabah	to proi	ı taşini
yarın	avriyo	taşteru
Buraya gel	Ela edho	Ela epae
Nasılsın?	Tikanis	İda kaniş
biraz	liğaki	miyaola

It is stated that there are many different Greek/Cretan words examples of which are given in the table.

According to Reginiotis [11]; The Islamized Cretans did not change either their language or their daily lives. Their language was Greek in the Cretan dialect. Although their names changed when they became Muslims, they adapted to the Greek language suffixes. For example, Selimis, Süleymanis, Çemalis, Nazifes, Fatmes. Some of the native Cretans who converted to Islam had Greek surnames (such as Abdül Kalimerakis, Mehmet Stafidakis). Those who came from Anatolia and maintained their Turkish surnames also adapted to the environment by adding the suffix ‘akis’ meaning ‘son’ to the end of these surnames. (Such as Muladakis, Alidakis, Hadzibekirakis).

Riginiotis, in his article, “has categorized the Turkish words added by the Cretans to their own dialect, many of which have Arabic origins, into three categories [11]”.

1. Words that are not used by Christians but only by Cretan Muslims: For example: Kadini (Woman), Hanumi (Lady), Ramazani (Ramadan), Qurani (Quran), Masalla (Mashallah)
2. Words that were also used by Christians, added to the Cretan dialect and many of which are still used in Crete today: For example: Kave (Coffee), Kavedzid (Coffee Shop), Çefi (Pleasure), Meraçi (Curiosity), Nazi (Naz), Hani (Han), Sevdas (Love), Dumani (Smoke), Odas (Room), Boğazi (Throat), Fesi (Fes), Kolayi (Easy), Amani (Aman), Lukumi (Turkish Delight).
3. Words that have entered the Cretan dialect but are no longer used or are little used today. For example: imani (iman), ibreti (ibret), sandzachi (Sanjak), duçani (shop), duçandzis (shopkeeper), salvari (shalwar), allir (Allah knows), bakalum (let’s see).

As can be seen, the Cretan Turks lost their ethnicity language, Turkish, and accepted Cretan as their language, but on the other hand, they brought a large number of Turkish words to their new language. In Bilgehan’s book [10], that have entered the Cretan language, most of them from Turkish words that were used in manis and in the Cretan language spoken in the past, some of which have also entered Greek there are more than 700 words. The pronunciation of some letters in Turkish is different. The letter ‘k’ in Turkish is mostly used as ‘ç’ (book: çitapi, Kemal: Çemalis, keyif: çefi, kebab: çebapi, clerk: çatipis etc.); The letter ‘y’ is pronounced as ‘j’ in many places (Elder: Jeros, Horse: Bejiri, Yoghurt: Jigurti, etc.), the letter ‘g’ is pronounced as ‘c’ in many places (Artichoke: Acinara. In the Cretan language, words with different structures and pronunciations than in Greek, and words of Turkish origin used in this language are common especially in Cretan manias. In addition to the old manis (Madinadas), some of these words are still used in the newly created manis today. (Love, lad, world, as is customary.)

Today’s Cretans still show special respect, though to a diminished extent, for their ancestral language, which they call “Kritika”. Among the inhabitants of Crete, there are still people with surnames of Turkish origin and probably of being Cretan Turkish origin. It is a fact that most of the Cretan Turks have lost their language and speak the Cretan language. Most likely, this is because the children of Turkish soldiers stationed in Crete, who were born when they married local women, had Cretan as their mother tongue. The influence of fathers and civil servant families in this unification was to ensure that a large number of Turkish words were established in the Cretan language. Today, Cretan as a pristine language is spoken in some isolated villages of Crete and by first-generation Cretan Turks, most of whom are over 80–90 years old, who came from Crete to Turkey with the population exchange and settled in Turkey. But it must be admitted that in a very short time there will be no more genuine(old) Cretan speakers in the world.

Since the subject of this study is limited to the identity, language and communication of Cretan Immigrants who migrated from Crete to Antalya and settled, the general situation of Cretans, reasons for migration, political, historical, economic and religious issues of Cretans are not mentioned. Identity, belonging, language and communication of Cretan immigrants, which are not directly encountered in the literature; In the context of multiculturalism, its concerns as a subculture are discussed.

#### Literature review

In literature, a large number of studies have been reviewed below for the theoretical framework of the research. Studies focusing on multiculturalism, migration, cultural diversity and education [17–20] and studies addressing the cultural and linguistic effects of migration and integration issues [6, 7, 21, 22] were reviewed. In literature, forced displacement, language use and ethnic identity [8, 16], interpersonal communication and acculturation in migrants [23], multiculturalism, identity and integration [9], the emergence of multiculturalism in Turkey [24], intercultural communication debates [25], multiculturalism in Turkey [26], multiculturalism and interculturalism in Eskişehir Circassians [27] were reviewed. A large number of studies in the literature [10, 12–16, 28–31] dealing with the island of Crete and Cretan immigrants from political, historical and economic aspects directly related to the subject of this research have been reviewed and reviewed. In the literature, there has been no direct study found regarding the identity, language, and communication concerns of Cretan migrants as subcultures in Turkey. The aim of the study is to contribute to social peace and tolerance by examining the identity, language, and communication perceptions and concerns of the Crete-origin community living in Türkiye as a subculture group, within the context of multiculturalism and intercultural communication. In this direction, the problem questions of the research can be as follows:

*How do Cretans living in Antalya define themselves*? *Do they experience anxiety in expressing their identity*? *What does Turkey mean to them*? *What does he know about his own identity and language*? *How do they communicate with the residents*? *What is their level of knowledge of Cretan*? *Do they see Cretan or Turkish as their mother tongue*? *Is Turkish or Cretan used as a language in their communication*? *Do they feel that they are subjected to pressure and assimilation in expressing their own identity and using their language*? *Do they worry about preserving*, *transmitting and losing their own identity and language*? The data obtained conducted in line with the purpose of the study were subjected to descriptive analysis in two main themes (categories) and three sub-themes. (See Method/Analysis of data)

## Materials and methods

In this study on identity, language and communication concerns of subcultures, qualitative research [32, 33] method, which is suitable for the research of cultural groups, was adopted as a method. Document, analytical and descriptive analysis methods were used in the analysis of the research data.

In the selection of “identity, language and communication concerns of subcultures” as the research subject, it is aimed to contribute to the research and studies on these problems, since the identity, linguistic and communicative problems that have become inextricable for both immigrants and countries with the excessive increase in migration mobility are effective.

The universe of the study is ethnic subcultures; the sample of the study consists of 18 Cretan participants who were reached by snowball sampling method in the first settlements of Cretan immigrants living as a subculture in Turkey in Antalya. In determining the participants to be interviewed from the Cretan immigrants in Antalya, the fact that Antalya is one of the cities where multiculturalism and cultural diversity are experienced the most; It has been effective for the Cretans of Antalya to be in contact with the Cretans in every region of Turkey and to been participation from every region in the activities by theirs.

The limitations of the study consist of 18 Cretan participants who were reached by snowball sampling method [34] in the first settlements of Cretans and Cretans living as a subculture in Turkey in Antalya.

The data sources of the research consist of written and visual materials [34] (documents) containing information on multiculturalism, identity, language and communication issues of the Cretan community, which is a subculture in Antalya, and preliminary interviews, observations and semi-structured in-depth interviews with Cretan immigrants.

### Collection of data

Scanning of written and visual materials (documents): Documents related to multiculturalism, dominant culture, subculture, identity, language and communication concerns of subcultures and Cretan immigrants in Turkey, which were determined within the conceptual framework of the research, were scanned and the data related to the research subject were noted.

### Fieldwork

The fieldwork was carried out between 08 May 2024 and 05 July 2024 by conducting telephone and face-to-face preliminary interviews, observations and semi-structured in-depth interviews with respected Cretans, local administrators and Cretan NGO managers living inside (Selimiye), Şarampol (Hamidiye), Boğazkent (Ahmediye) and Kadriye, which are the first settlements of Cretans in Antalya. See S1–S6 Figs. (In order to protect personal data, there is no participant image in the photos, and the image of the person who appears belongs to the researcher. In all interviews, people were informed about the subject of the research, its objectives, the confidentiality of personal data, and the freedom of participation, and an “informed consent form” was obtained from the participants.)

Preparation of interview questions: After the literature review conducted in line with the subject and objectives of the research, a draft of semi-structured questions has been created by the author. In the preliminary interviews, the opinions of prominent Cretans, local administrators and Cretan NGO managers have been taken regarding the determination of the participants and about the draft question. Some corrections have been made to the questions. After receiving expert opinions about the questions, the questions were finalized, and an “Interview Form” was prepared. (*However*, *during the interview process*, *not only were the questions written in the interview form adhered to*, *but also the interviews were developed in depth with new questions according to the flow of the interview*.) An “Ethical compliance report” was received from the relevant authority for the study. (Akdeniz University Rectorate Social Sciences and Humanities Scientific Research and Publication Ethics Board. Ethics committee date: 07.05.2024, Number: 09, Decision No: 244)

Identification of participants and interviews: In order to contribute to the validity and reliability of the data in the determination of the participants, the opinions of respected Cretans, local administrators and Cretan NGO managers who stand out in the region; age, profession, recognition, respectability, educational status, and knowledge about the research subject were taken into account according to the characteristics of the people. To obtain accurate, reliable, and comprehensive data, participants have been assured of the protection and confidentiality of their personal data in order to ensure that they feel safe while sharing their experiences. Information on the demographic structures of the determined participants is shown in the table (Table 2).

**Table 2:** Demographic information of the participants who make up the sample.

Participants	Gender	Education Status	Residential area	Age	Status	Preliminary interview	Interview
P1	Male	University	Side/Selimiye	69	Tourism Sector	June 01, 2024	Jun 07, 2024
P2	Male	Middle School	Side/Selimiye	65	Tourism Sector	June 01, 2024	Jun 07, 2024
P3	Male	Middle School	Side/Selimiye	68	Retired	June 01, 2024	Jun 07, 2024
P4	Male	University	Side/Selimiye	84	Retired Captain	June 01, 2024	Jun 07, 2024
P5	Male	Middle School	Side/Selimiye	64	Retired	June 01, 2024	Jun 07, 2024
P6	Male	Primary School	Kadriye	94	Farmer/Retired	May 25, 2024	Jun 01, 2024
P7	Male	Primary School	Boğazkent/Ahmediye	88	Trader/Farmer/Retired	May 25, 2024	Jun 01, 2024
P8	Male	Middle School	Boğazkent/Ahmediye	64	Farmer/Livestock	May 25, 2024	Jun 01, 2024
P9	Female	High School	Kadriye	62	Private Sector/Retired	May 25, 2024	Jun 01, 2024
P10	Female	Middle School	Boğazkent/Ahmediye	53	Housewife	May 25, 2024	Jun 01, 2024
P11	Female	University	Şarampol/Hamidiye	75	Civil Servant/Housewife	May 14, 2024	May 28,2024
P12	Female	High School	Şarampol/Hamidiye	58	Housewife	May 21, 2024	May 28,2024
P13	Female	High School	Şarampol/Hamidiye	54	Housewife	May 21, 2024	May 28,2024
P14	Female	Middle School	Şarampol/Hamidiye	62	Retired	May 21, 2024	May 28,2024
P15	Female	Middle School	Şarampol/Hamidiye	72	Housewife	May 21, 2024	May 28,2024
P16	Female	Middle School	Şarampol/Hamidiye	68	Housewife	May 21, 2024	May 28,2024
P17	Male	High School	Kadriye	27	Trader	May 25, 2024	Jun 01, 2024
P18	Male	High School	Şarampol/Hamidiye	58	Journalist	May 08.2024	July 05, 2024

### Analysis of data

Document, thematic and descriptive analysis techniques, which are qualitative research methods, were used to analyze and interpret the data in the research. The data obtained from the literature review, observations and interviews conducted in line with the subject and purpose of the study to the following categories and themes to the established criteria (Table 3).

**Table 3:** Categories, themes and questions determined for data analysis.

**Category** ***Identity and belonging*** **Themes** *• Self-definition*, *belonging*, *and identity concerns*	**Questions** • *What is the identity you define yourself with*? • *What do Turkish identity*, *Turkish citizenship and Turkish language mean to you*? • *Do you feel uneasy about expressing your identity openly*? • *Do you feel anxiety about losing your identity*?
**Category** ***Language and communication*** **Themes** *• Mother tongue phenomenon*, *level of knowing the mother tongue (Cretan)*, *desire to learn and speak*, *and concerns* *• Language*, *communication and concerns*	**Questions** • *What is your mother tongue*? • *What is your level of knowing Cretan*? • *What is the language you use among family members and Cretan neighbors*? • *If you have communication with Cretans in Crete*, *in which language do you communicate with them*? • *Are there any Cretans in Turkey who do not speak Turkish or know little*? • *Do you need to learn and speak Cretan*? • *Do you feel any restrictions*, *pressures*, *uneasiness in speaking Cretan*? • *Are you concerned about the preservation and loss of the Cretan language*? • *Does the fact that the official language and language of instruction is Turkish pose a problem to keep your identity and language alive*? • *Do you make a connection between knowing and keeping the Cretan language alive and protecting your identity*?

The data obtained from the data sources of the research; It was summarized according to the determined themes and interpreted by subjecting it to thematic and descriptive analysis. To ensure the orijinal representation of experiences, to strikingly reflect participants’ perspectives, to enrich the narrative, to enhance the reliability of the findings, and to provide qualitative interpretations, numerous direct quotations from participants have been included. In addition, participant narratives and observational insights have been combined to add depth to the analysis and improve comprehensiveness. The depth of the study has been increased through the thematic organization and the use of participatory voices, and insights into the identity, language and communication practices of Cretan immigrants have been presented.

## Results

In this section the data obtained for the purpose of the study was summarized in two main themes (categories) and three sub-themes and subjected to thematic descriptive analysis.

A large number of direct quotes from the participants were included in order to ensure that the experiences were represented in an authentic way, to reflect the views of the participants in a striking way, to enrich the narrative, and to increase the reliability of the findings.

### Identity and belonging

In this category, in order to evaluate the identity, belonging and concerns related to these that Cretans attribute to themselves, theme was focused on: “*Self-definition*, *belonging*, *and identity concerns*”.

#### Self-definition, belonging, and identity concerns

In this context, the participants were asked the following questions: “What is the identity you define yourself with?”, “What do Turkish identity, Turkish citizenship and Turkish language mean to you?”, “Do you feel uneasy about expressing your identity openly?”, “Do you feel anxiety about losing your identity?” As a result of the answers, comments and observations made by the participants, the following findings were reached regarding identity and belonging. Of the 18 participants, 9 defined themselves as “Turkish of Cretan origin”, 2 participants as “Cretan immigrants”, 2 participants as “Cretan Turkish citizens”, 3 participants as “Turkish” and 2 participants as “Cretan”. It is seen that the majority of the participants see Turkey as their homeland with the awareness that Turkey has accepted them under difficult conditions and that they want and adopt Turkish citizenship of their own free will. Some of the participants stated that they were flinched to say that they were “Cretan” in the following periods, especially when they stated that they were “Cretan” in their childhood and early youth, because they faced reaction and exclusion. As a researcher observation, it is understood from the discourse and behavior of some of the participants that; For them, they see being “Turkish” as a higher identity and being “Cretan” as a sub-identity below it. Most of the participants have stated that they are worried about losing their identity due to the difficulty in transferring Cretanity to the next generations. Some of the views of some of the participants on identity and belonging are given below as direct quotes.

“I am a Turk because my generation is Turkish. I am the third generation of first-generation Cretan immigrants. I’m proud of who I am, and I don’t mind saying it openly. The first generation was excluded, but then, after reflecting the benefits of Cretan culture, this idea was abandoned, because of the impression given by all kinds of cultures…” (P4)

“I feel like I’m Cretan. I wonder what my family has been through in its nearly 250 years of Cretan life, what kind of social change it has experienced… Turkish identity, like the identity of other countries, is to be entitled to receive Turkish citizenship… I had a certain uneasiness about revealing my identity openly in my childhood and early teenage years, and I don’t feel uneasy anymore… My concern is that these good feelings will disappear after a generation or two. I feel a responsibility to pass on that feeling to new generations.” (P1)

### Language and communication

In this category, two themes are emphasized: “Mother tongue phenomenon, level of knowledge of the mother tongue (Cretan), desire to learn and speak, and concerns”, “Communication language and concerns.” In this direction, the participants were asked, “What is your mother tongue? (Turkish/Cretan/Other)”,”What is your level of knowing of Cretan?”, “What is the language you use among family members and Cretan neighbors?”, “If you have communication with Cretans in Crete, in which language do you communicate with them?”, “Are there any Cretans in Turkey who do not speak Turkish or know little?”, “Do you need to learn and speak Cretan?”, “Do you feel any restrictions, pressures, uneasiness in speaking Cretan?”. “Are you concerned about the preservation and loss of the Cretan language?”, “Does the fact that the official language and language of instruction is Turkish pose a problem to keep your identity and language alive?”, “Do you make a connection between knowing and keeping the Cretan language alive and protecting your identity?”. The following findings were obtained from the answers, opinions and observations given to the questions.

#### Mother tongue phenomenon, level of knowing the mother tongue (Cretan), desire to learn and speak, and concerns

Findings on the theme of “mother tongue”; the following findings were obtained from the answers given to the question “What is your mother tongue?” participant opinions and from our observations.

The participants who only speak Turkish as their native language are 60%; those who speak both Turkish and Cretan as their native language are 20%; those who express their native language as only Cretan are 15%. The most remarkable finding is that some participants, despite stating that their ancestors came from Crete, and they did not know any Turkish at that time, and that they only spoke Cretan in the family, they stated that their mother tongue was “Turkish”. Apart from these, three participants answered the question “What is your mother tongue?” as “I know Turkish and little Cretan”. Some of the participants’ views on mother tongue are given below as direct quotes:

“I know Turkish and a little Cretan” (P4), “Since our roots are Anatolian, Turkish is our only language… I know Turkish and Cretan” (P3), “Turkish” (P2) “Turkish (a little Cretan)” (P1), “Turkish” (P5), “Cretan, I learned Turkish later, I improved it at school…” (P6), “My mother tongue is Cretan” (P7), “Turkish” (P17), “Turkish” (P15), “Turkish, Cretan” (P14), “My father’s Cretan-Turkish; My mother tongue is Turkish” (P13), “Turkish” (P12), “My mother tongue is Turkish” (P11), “Turkish” (P10, P18), “Turkish and Cretan; I say I have two mother tongues.” (P9).

*Level of knowing of Cretan and Turkish*. The following findings were obtained from the answers given to the question “What is your level of knowing of Cretan language (you, your family)” and “Do you know any Cretans in Turkey who do not speak Turkish or know little?” participant opinions and from our observations.

Although of the participant P5 stated that his parents knew very little Turkish and that they always spoke Cretan in the family, it is contradictory that he said that his “mother tongue” was Turkish. According to the statements of the participants, the family members who were the first to come from Crete and are not alive now have a Cretan mother tongue and either do not know Turkish at all or know very little; It is understood that the children of the first arrivals learned Turkish at school and became bilingual, but over time, Turkish became the dominant language and began to forget Cretan. It is seen that the children of those who know little Cretan or are on the verge of forgetting it understand a word or two in Cretan or do not know Cretan at all and speak Turkish. It is understood from the opinions of some participants who were able to speak a little bit of Cretan in their childhood that the fear of exclusion related to the language spoken by their ancestors or the language they themselves experienced during their childhood has led them to turn towards Turkish, which is the dominant language, and as a result, Cretan has remained in the background and has not been taught or transmitted. Participants, in the process when Cretan weakened and the use of Turkish increased; It is stated that while men learn Turkish earlier, women learn Turkish late. In addition, it is understood from the participant statements that for those who know Cretan, speaking Cretan is normal, natural and sincere; It was seen as forced to speak Turkish. It was possible for them to adopt Turkish later and in lower lineages. Some of the participants’ views on the level of knowing of Cretan are given below as direct quotes:

“I speak little Cretan; our upper family spoke only Cretan. In the first generation, there were people who did not speak Turkish. (A little Turkish was spoken.)” (P4), “I can speak Cretan, my father, my grandmother, my mother, my whole family spoke it very well. Now there are very few who know. In the past, 50–60 years ago, there were many people who did not speak Turkish…” (P3), “I had a low level of knowledge of Cretan, In Cretan knowing my father was good, my grandmother was very good. (It is lost from generation to generation.)” (P2). “I can speak very little Cretan (on a word-by-word basis), there are still people in my family who can speak Cretan at an intermediate level. My upper family could only speak Cretan… There is no one around me who does not speak Turkish at the moment, the language of communication is Turkish.” (P1), “I know, I speak Cretan. When I go to Crete, after 3 days I speak fluently… In the past, our elders, grandfathers, mothers and fathers knew very little Turkish, but now there are no these Cretans left…” (P5).

*The need to learn and speak Cretan*. The following findings were obtained from the answers given to the question “If you do not know Cretan, do you need to learn and speak Cretan?”, participant opinions and from our observations.

Participants who did not know Cretan or knew very little have been stated that they felt the need to speak Cretan and that they wanted to learn it very much. They have also been stated in their answers that they especially wanted their children and descendants to learn. In particular, the president of the Antalya Cretans Association, one of the participants, have been stated that they opened a “Cretan course” twice due to this need, but they were not successful, and stated that every language in the world should be protected and kept alive, and therefore courses for all subcultural languages should be opened not only for Cretan but also for all subcultural languages by the government.

Some of the participants’ opinions regarding the need to learn and speak Cretan are directly quoted below.

“We talk Cretan as much as we can, I didn’t take educated for this.” (P4), “Yes (I need)” (P3), “Of course (I need)” (P2), “Yes, I do. For this reason, we are planning to open a Cretan language course at the Side Crete Culture House. (P1), “I know Cretan…” (P5), “… I know, those who don’t know should learn.” (P6). “I know, and my kids want to learn.” (P7), “I know, I speak, I need it so that it is not forgotten… In the past, Cretan tales were told for weeks.” (P8), “I speak less Cretan and understand more, I would be very happy if I spoke more, I need it.” (P16), “I am interested because I am curious about the language of my ancestors.” (P15), “I like it if I speak Cretan, I feel the need to speak.” (P14), “I’d love to, but I’d like to learn.” (P13), “I understand Cretan, but I can’t speak it. I’d love to talk.” (P12).

*Restriction*, *pressure*, *or uneasiness in speaking Cretan*. The following findings were obtained from the answers given to the question “Do you see or feel any restriction-pressure or uneasiness in speaking Cretan?”, participant opinions and from our observations.

Participants have been stated that their parents and grandparents (Superior generation family members) experienced pressure and uneasiness rather than restriction in speaking Cretan. In addition, some participants have been stated that they experienced the same psychological pressure and uneasiness in their childhood and youth. They have been stated that their superior generation family members have been excluded, especially in the early days; They have also been stated that they were subjected to psychological pressure and restriction, especially when they spoke Cretan at school. The young Cretans have been stated that they did not experience such restrictions and uneasiness because they did not already know Cretan, but they knew from the anecdotes and memories of their superior generation family members that this had happened. Most of the participants have been stated that there is no such pressure or restriction today, and there is no uneasiness.

Some of the participants’ views on the issues of restriction, pressure or uneasiness in speaking Cretan are given below as direct quotations.

“At first, it was not restraint, but uneasiness, strangeness, and they still get angry sometimes.” (P7), “There was no pressure to speak Cretan. It happened in Kadriye.” (P8) said, “What happened to our parents has been repression. Our aunt’s daughters saw it(repression). My mother went to school, she spoke Cretan, she couldn’t speak Turkish, so they had been hit him with a ruler…” (P16), “I don’t feel it right now. Our elders feel it in the past…” (P15), “When my parents were gathering grass in Boğazak, the locals (Turks) used to say, ‘The cows have come, run away’. There were such problems, we and the next generation did not experience them.” (P14), “My elders have experienced it in the past, but now we have no restrictions at all.” (P13), “I didn’t feel any pressure. My family wasn’t pressured either. But as a child, I had a fear of being ostracized.” (P12), “Not the restriction and pressure, but my parents were a little feeling nervous, they were excluded.” (P11), “No, I was not pressured or restraint” (P10).

*Concerns about protecting*, *keeping alive and losing Cretan language*. The following findings were obtained from the answers given to the questions about protection, survival and anxiety about losing the Cretan language, the opinions of the participants and from our observations.

Although the majority of the participants did not see Cretan as their mother tongue, they have been expressed concern about the disappearance of Cretan. The majority of the participants have been stated that it was not a problem for the language of instruction in schools to be Turkish, but they wanted Cretan lessons to be held during the school period. All of the participants expressed their deep concern about the disappearance of the Cretan language and have been stated that Cretan courses can be opened by the authorities, just like the English-Russian courses given by the Ministry of National Education (Ministry of National Education) or municipalities. All of the participants have been stated that the Cretan language should be protected, that they were concerned about its loss, and that they organized Cretan courses in the associations they established. They have been stated that they want young people, especially those who do not know Cretan at all, to come to these courses and learn Cretan.

It’s about this theme; The answers given by the participants to the questions “Are you worried about the loss of Cretan Language?” and “Does the fact that the language of instruction is Turkish pose a problem for your cultural identity and the protection and survival of Cretan?” and some of their opinions are given below as direct quotes:

“No (I don’t feel worried about my mother tongue being lost). No, it is not a problem (that the official language and the mother tongue is Turkish).” (P4, P17), “I’m not worried but I hope that Cretan courses will be opened so that Cretan will continue and not be forgotten.” (P3), “Before the metropolitan law, we elected our own headman and mayor and lived according to our own customs and traditions in accordance with the laws of the Republic of Turkey, but now this freedom is partially restricted.” (P2), “I am experiencing anxiety, children do not know any (Cretan), they must learn, our culture must continue…” (P6), “It’s not a problem, but Cretan language and Cretan culture need to be preserved.” (P7), “I’m sorry, I’m worried, I’m trying to teach… It would have been great if children had been taught Cretan in addition to Turkish, but there are no more expectations now.” (P8), “Of course, we are worried, let the Cretan language not disappear, let our culture, our language come to the surface, let us be visible… In fact, if there was a lesson in Cretan and Cretan culture, I would want it in schools…” (P16). “I see my mother tongue as Turkish, but I don’t want Cretan to disappear. In fact, all the languages used in the world should not disappear. I think language is the cultural richness of the world…” (P18).

#### Language, communication and concerns

*Language used within the family and among Cretan neighbors*. The following findings were obtained from the answers given to the question “What is the language used in communication between family members and Cretan neighbors?”, participant opinions and from our observations.

It is stated that the elders in the family speak both Cretan and Turkish among themselves, those under the age of 60 mostly communicate in Turkish and sometimes in Cretan because they know little Cretan, and those under the age of 30 speak only Turkish because they know almost no Cretan. In addition, it has been stated that those who speak Cretan feel comfortable, happy and proud when they speak Cretan among themselves. These positive feelings were also seen in the observations of the researcher, and it was witnessed that they were proud to speak Cretan, especially on special days and meetings between neighbors, and that they sang manias in Cretan with enthusiasm and excitement. Examples of manias said by P6, P9 and P11 in these conversation and mania telling activities are given below (Table 4).

**Table 4:** Examples of manias sung in Cretan by P9, P6 and P11 of the participants.

*Cretan (Kritika)*	Turkish
*Krìti dhen tha se ksehàsune i Kritiçì sta ksèna*, *Krìtimu su t’orkìzome sti màna pu mas yèna*	Girit seni unutmazlar, Giritliler gurbetlerde, Yemin ederiz Girit’im bizi doğuran ana üzerine.
***English***	Crete, they will not forget you, the Cretans in the expatriates, We swear by the mother who gave birth to us.
*Op uça pào tha vasto hama apto Psibriti* *Na to skarpizo na yani olos o kozmos Kriti*	Gideceğim tüm yerlere Psiloriti’den toprak taşıyacağım Onu seveyim ki her yer tüm dünya Girit olsun.
***English***	I will carry earth from Psiloriti to all the places I go, May I love him so that everywhere and the whole world will be Crete
*Ahita perazmena çahita parezumena*, *Naksa mueyernune toplunun naimera*	Ah geçmişim, ah geçmiştekiler, Ne olurdu senede bir kere dönselerdi
***English***	Oh, my past, oh those in the past, What would happen if they returned once a year?

Some of the respondents’ views on the language used in communication between family members and their Cretan neighbors are given below as direct quotes.

“For me Turkish, how those but Cretan speak to each other beautifully (in Cretan).” (P13), “We speak Turkish, but when our old Cretans meet, they speak Cretan.” (P12), “I speak Turkish with my parents, but the neighbors always spoke Cretan. Cretan was spoken secretly from the young people… .” (P11), “My parents spoke to each other in Cretan, we siblings spoke Turkish…” (P10), “We used to speak Turkish now, but we used to speak Cretan and Turkish during the parental period. The first generation did not speak Turkish…”, (P9), “Turkish” (P17).

*Communication with Cretans living in Crete and the language used*. The following findings were obtained from the answers given to the question “If you have communication with the Cretans in Crete, in which language do you communicate with them?”, participant opinions and from our observations.

According to the participant responses, it is understood that Cretans in Crete and English communicate with them. There are also participants to Crete and Crete who do not have any contact with Cretans. It was stated that none of the participants had any relatives or acquaintances in Crete. The striking finding here was that all of the participants longed for Crete, even though they had no relatives or acquaintances left in Crete. They were stated that when they went to Crete due to the change in the spoken language of Cretan over the years, the Cretan people were surprised because they spoke old Cretan with the people of Crete, and they were immediately asked which village they were from in Crete. They also have been stated that excursions to the island of Crete were organized, but young people did not participate in the trips. It has been stated that when they went to Crete, due to changes in the spoken language over the years, they spoke old Cretan with the locals, the Cretan people were surprised by these. They have been stated that the Cretans immediately asked which village they came from. It has also been mentioned that excursions to the island of Crete were organized, but the young people did not participate in these trips.

Some of the participants’ views on communication with Cretans living in Crete and the language used are given below as direct quotes.

“We speak Cretan” (P4, P3), “We speak English, and very little Cretan” (P2), “We have communication. We communicate using 3 languages.” (P1) “In Crete, we usually speak Cretan, and we get along very well. They say, ‘What village do you come from?’ because we speak the language that was spoken 100 years ago, they like it.” (P5), “We don’t have much communication” (P6, P8, P14, P11, P10, P17, P12).

*Establishing a connection between preserving the Cretan language and maintaining identity*. It’s about this theme; The following findings were obtained from the answers given to the question “Do you establish a link between knowing and keeping the Cretan language alive and protecting your identity?”, participant opinions and from our observations.

Three participants didn’t answer this question. Some of the participants have been started the question by saying that they are “Turkish”, and that Turkish is their mother tongue, then have been stated that the concepts of culture, identity and language are related to each other and have been stated that Cretan, which is a part of Cretan culture, should not be forgotten. Some of the participants, state that Cretan language should live in order not to forget Cretan identity because Cretan language and Cretan culture are one and interconnected. All of the participants who answered the question stated that there is a link between language and identity. Some of the participants’ views on the issues of establishing a link between keeping the Cretan language alive and preserving the identity are given below as direct quotes.

“It will be very nice to keep our past alive by organizing events and seminars.” (P3), “Yes, I connect. Therefore, I believe that urgent work and efforts are needed.” (P1) “Of course, I connect, language is very important, it has to try to teach.” (P7), “Language and identity are linked. There is a high bond…” (P16) “I’m connecting (between language and identity)…” (P15), “Of course, I’m connecting. Culture, language, identity are one. Cretan language and Cretan cultural identity are one…” (P14), “I would love to live knowing Cretan, when I meet those who know, my soul goes to Crete, and my Cretan identity rises in an instant.” (P13), “Of course, it’s all connected. We have tried to preserve all of them until our generation, but it is a pity that they are slowly disappearing in the generations after us.” (P12)

As a result: Due to the weakening of the Ottoman rule on the island of Crete and the turmoil that occurred on the island, migration to Anatolia began. Cretans, who came to Anatolia with forced migration and exchange, were settled especially in the coastal regions of Turkey. One of these regions is Antalya, one of the cities where multiculturalism is the most intense in the world. Of course, as in every region where multiculturalism is experienced, there have been intercultural concerns, uneasiness and discussions among Cretans and natives in Antalya. As Manea puts it, “Within individuality, there can be a conflict between how they perceive themselves and how society evaluates them” [35]. While the Cretans on one hand want to preserve, sustain, and pass on their own culture, identity, and language to future generations, they have also simultaneously tried to adapt to the culture, language, traditions, and customs of the country they live in. From the narratives and our observations, it is clear that the biggest concern is related to language and identity. It was observed that all participants, from the oldest to the youngest, interiorized and take ownership Turkish citizenship and identity, but were also proud of their Cretan identity. The same is true for language. It is among the notable findings that the participants expressed that the Cretans who first came to Anatolia did not know Turkish while stating that their mother tongue was Turkish, that there is a strong solidarity among themselves, that they desire to use Cretan in their communication, that they believe Cretan is a unique language different from Greek, and that they expressed their excitement at a song sung in Cretan.

## Conclusions and discussion

In this study, which investigates the identity, language, and communication concerns of subcultures, the Cretan immigrants in Turkey are examined as a subculture. Antalya, one of the provinces where multiculturalism is experienced most intensely in Turkey, has been determined as a sample.

The results obtained from thematic and descriptive analyses of written and visual sources, participant narratives, and observational insights that constitute the data source for the research are as follows:

It can be said that the Cretans in Antalya are preserving the cultural elements specified in the Convention for the Safeguarding of Intangible Cultural Heritage [36] related to their identity. However, there is concern about transmitting these cultural elements to future generations and the risk of gradual disappearance. The highest concern is about language, and they believe that their language must be kept alive in order to protect their identity. In this study, it was determined that although the majority of the participants accepted Turkish as their mother tongue, their beliefs that Cretan should be protected, kept alive and transmitted were one of the most being sensitive issues.

Participants have been expressed concerns about the preservation of the Cretan language while emphasizing their aspirations for the future and their efforts to sustain their heritage. The concept of mother tongue at Crete immigrants, language usage, and the emphasis on Cretan language courses highlight a vital aspect of cultural and linguistic preservation. Concerns regarding the preservation and transmission of the Cretan language resonate with the broader issues faced by many immigrant communities. Particularly noteworthy is the participants’ emphasis on the relationship between language preservation and the maintenance of identity. The results effectively demonstrate that for many participants, knowing and speaking Cretan is an integral part of their sense of self and cultural continuity. This connection can make an important contribution to the debate on minority languages and cultural identity.

The fact that the study was limited to 18 participants may be seen as insufficient to draw generalized conclusions about the identity, language and communication of Cretans in Turkey. Although a large sample size is not mandatory in qualitative research, it can be thought that a large number of participants can increase the richness of the data and provide more diverse perspectives. However, it is thought that this deficiency can be eliminated by the fact that the Cretans of Antalya, who constitute the sample of the study, are in contact with the Cretans in every region of Turkey and Crete, that there is participation in their activities from every region, and that Antalya is one of the cities where multiculturalism and cultural diversity are experienced the most. Additionally having participant are managers from the Crete civil society organization and respected individuals in the region, as well as individuals married to members of the dominant culture, can contribute to the richness of the data and the generalizability of the results.

The balance between the rights of subcultures and their responsibilities to the host community is the most important issue to be considered. The countries’ approach to positive integration rather than assimilation, the potential benefits of multiculturalism, the need for a harmonious coexistence that respects all identities can promote understanding and tolerance between communities. With this proactive stance, on the one hand it is intended to make the study effective and increase its credibility, and on the other hand, to encourage scientists to further investigate the nuances of cultural identity, language and integration in similar contexts. The findings revealing identity complexity among the immigrants from Crete, highlight how participants navigate their identities in relation to Turkish citizenship and Cretan heritage. Insights into their concerns about losing their identity are projected to provide a deep understanding of the challenges minority communities face in preserving their cultural heritage.

There are many scientific studies on the subject of this study (migration, identity, multiculturalism, integration, assimilation, intercultural communication and language).

From the results of the study [37], which deals with the sociological view of Cretan immigrants in Antalya; While the level of Cretan language proficiency among Cretans, the transmission of the language and the concerns of survival coincide with the results of this study, the fact that the majority sees Cretan as their mother tongue is contradictory. Because in this study, the majority of the participants stated that their mother tongue was Turkish. (*Although their grandparents said they didn’t speak Turkish*.) In another study [38], it was concluded that Circassian youth living in Kayseri strongly embrace their identity as a subculture, but this embracement varies according to gender, and that although the identity is felt very strongly, participation in activities that could contribute to the preservation and continuity of the identity is low. In this study, there was no difference in feeling and owning Cretan identity according to gender. Although the rate of taking part in Cretan associations and becoming a member is low, it has been determined that the desire to participate in cultural activities for the protection and transmission of Cretan identity and language is very high.

Today, there is almost no culture that does not interact, and multiculturalism has become the most important phenomenon of all societies. The perception that the different cultures, identities and languages within the country can lead to the degeneration of the dominant culture’s own culture and language and harm national unity; On the other hand, the actions that can be taken with the concerns of immigrants about the loss of their own culture, identity and language, protection and transfer can cause conflicts. Instead of assimilating or destroying different identities, languages and cultures as a result of mismanagement of the migration and integration process, efforts should be made for positive integration. Multiculturalism (identity, cultural, linguistic diversity and difference within the country); It should be seen as a factor that increases solidarity, cohesion, desire to live together, better understanding and tolerance of each other and enriching human creativity, and scientific studies to be carried out in this direction should be supported.

## Supporting information

S1 Fig Şarampol Cretan Culture Hause and Cretan Women’s Counselling Centre. (TIF)

S2 Fig Şarampol Cretan Culture Hause. (TIF)

S3 Fig Cretan Identity and documents who have become Turkish citizens. (TIF)

S4 Fig Side (Selimiye) Culture Hause. (TIF)

S5 Fig Side (Selimiye) Culture Hause. (TIF)

S6 Fig Side (Selimiye) Culture Hause. (TIF)
