# Supplementary figures and images for: Identity, language and communication concerns of subcultures: The case of Antalya Cretans
Source: PLoS One. 2025 Jan 6;20(1):e0314543. doi: 10.1371/journal.pone.0314543 (PMC11703064; doi:10.1371/journal.pone.0314543)

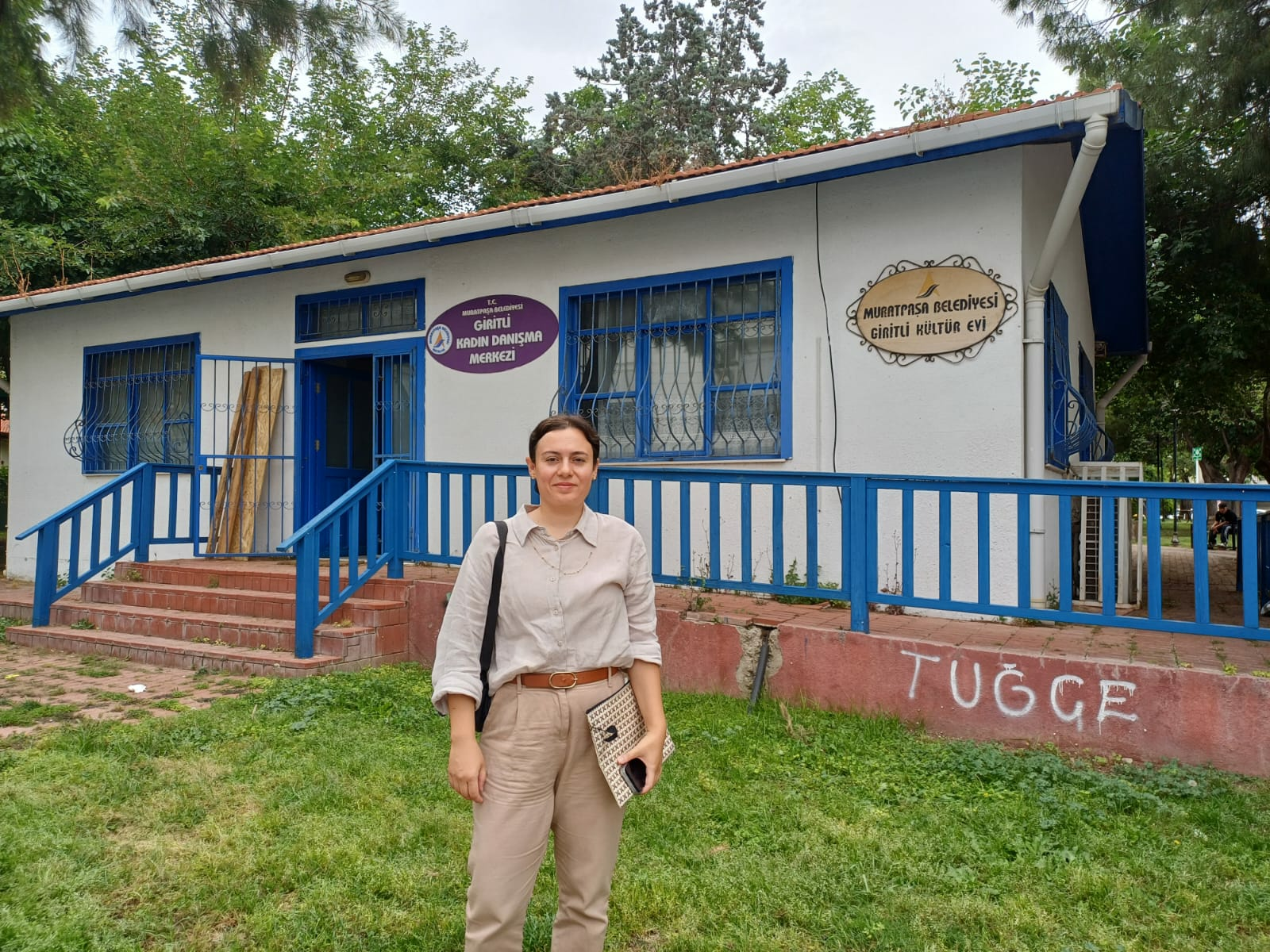

Supplement: S1 Fig — (TIF) [file pone.0314543.s001.tif]

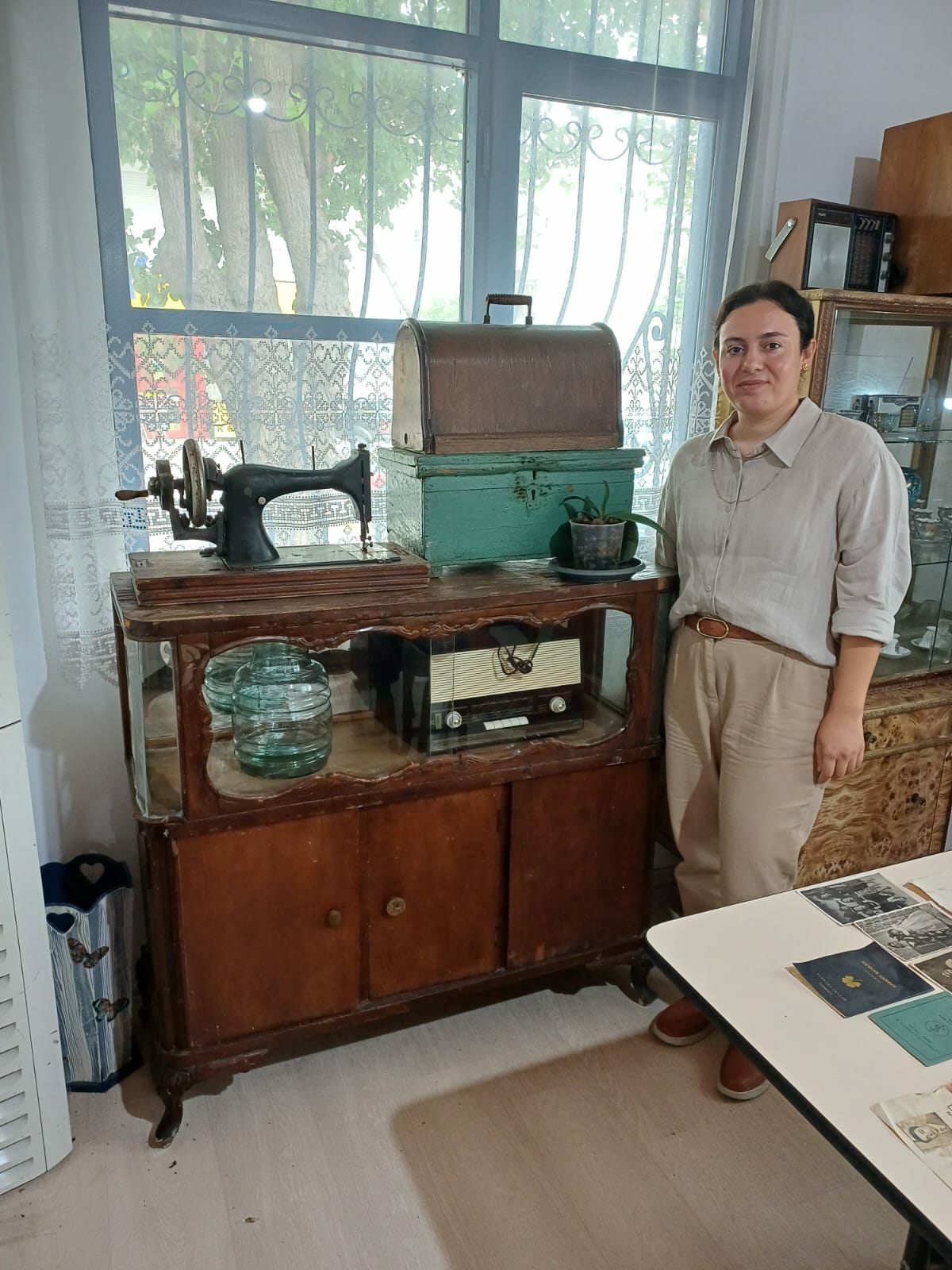

Supplement: S2 Fig — (TIF) [file pone.0314543.s002.tif]

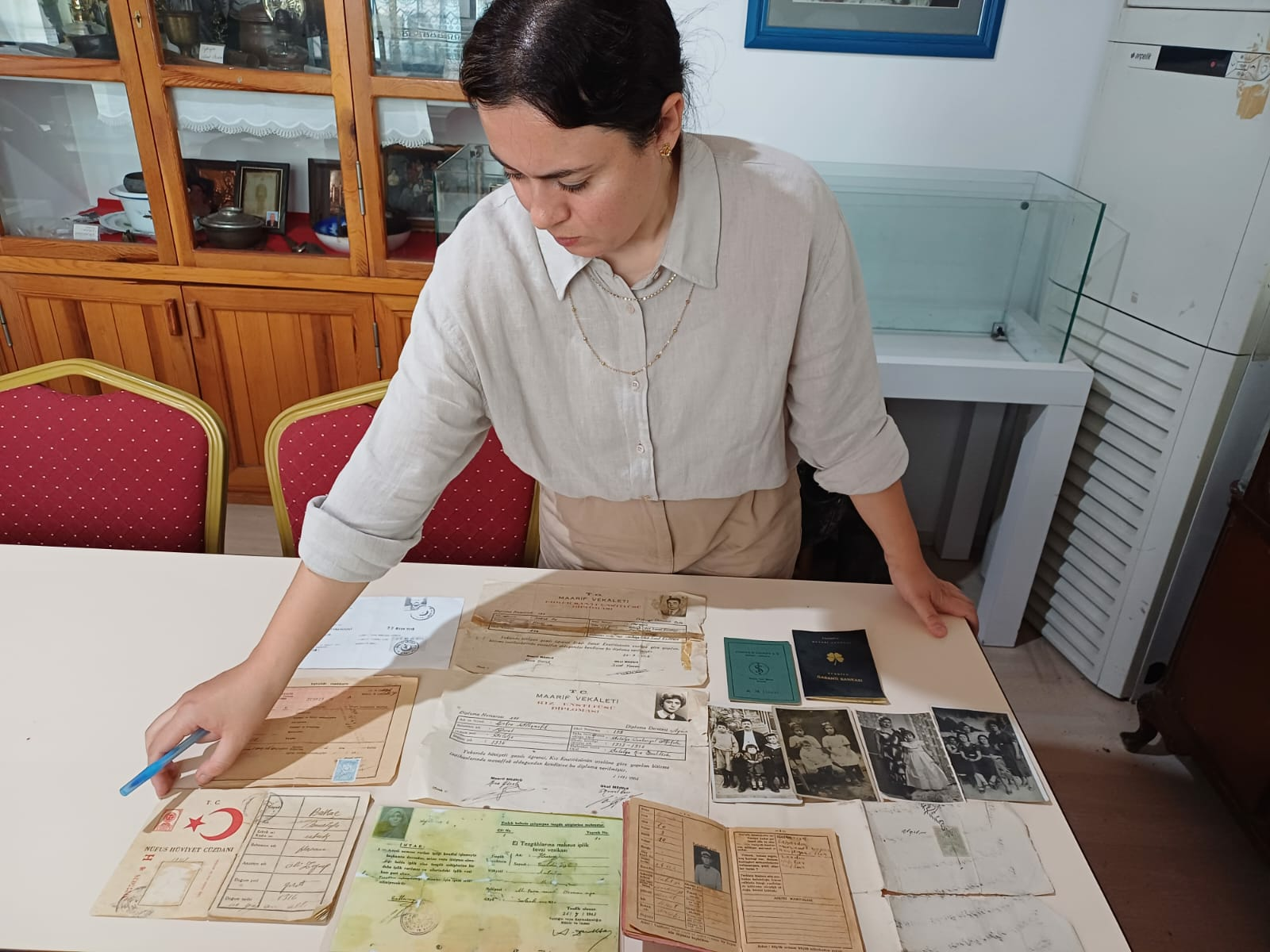

Supplement: S3 Fig — (TIF) [file pone.0314543.s003.tif]

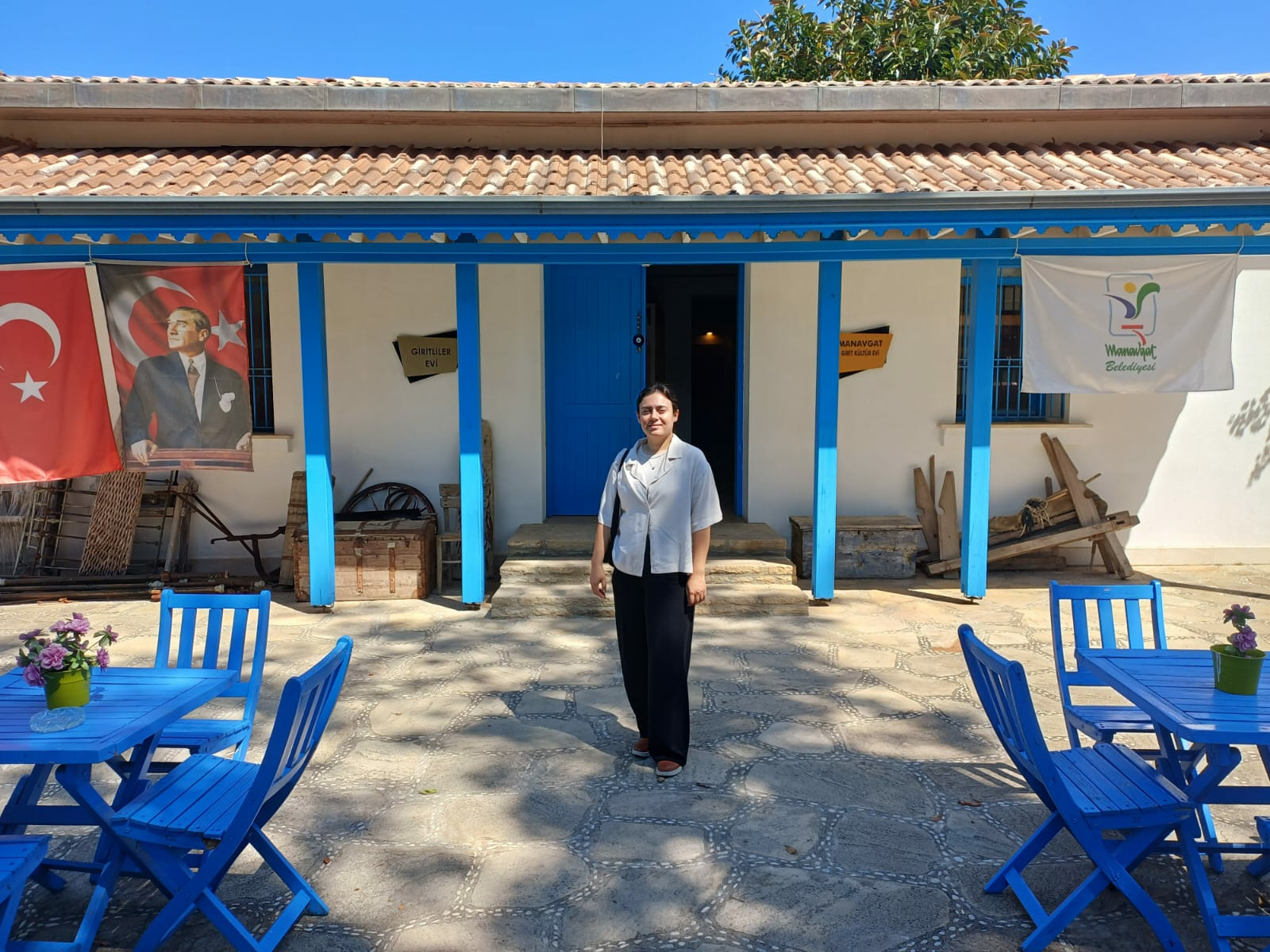

Supplement: S4 Fig — (TIF) [file pone.0314543.s004.tif]

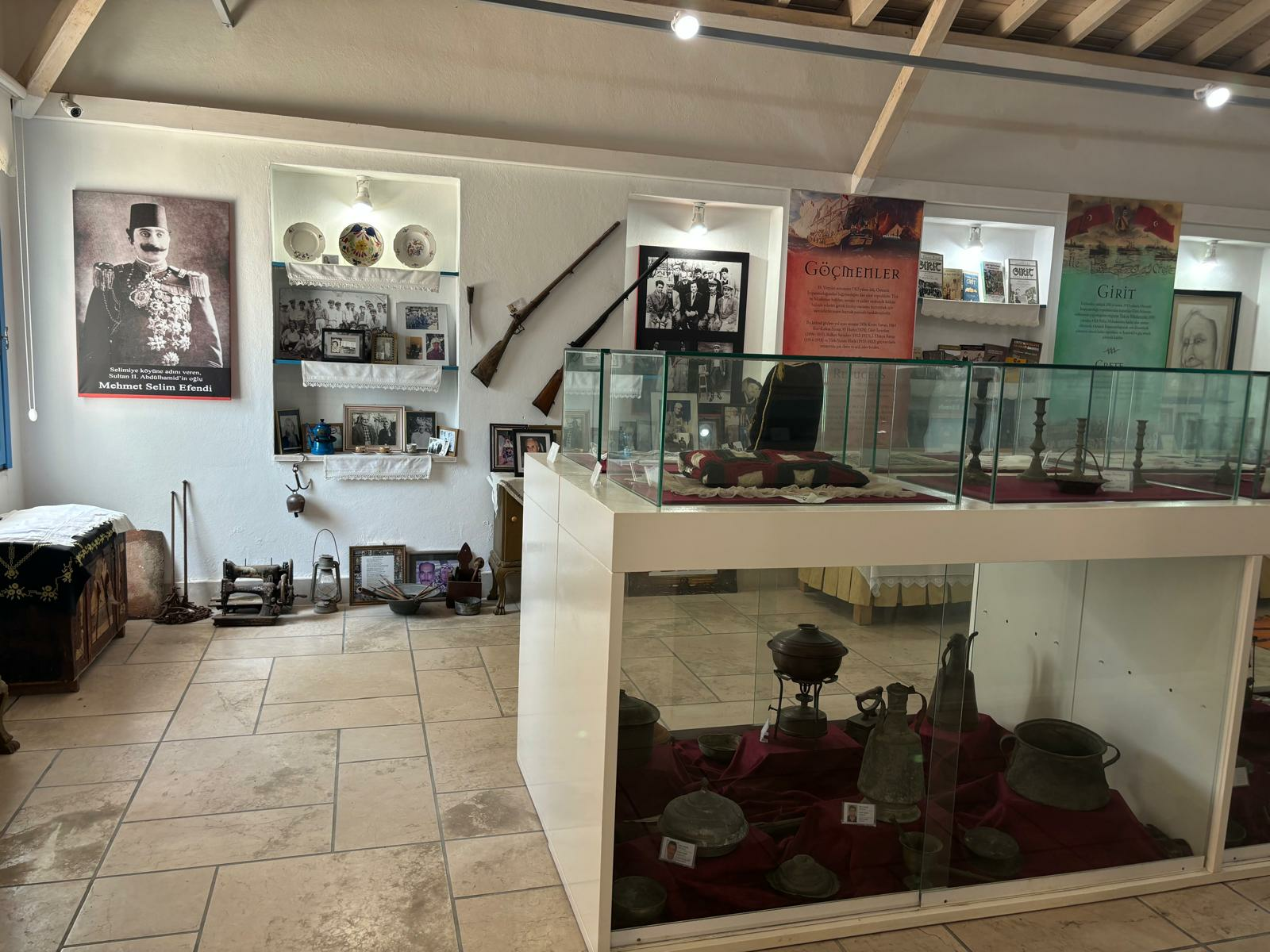

Supplement: S5 Fig — (TIF) [file pone.0314543.s005.tif]

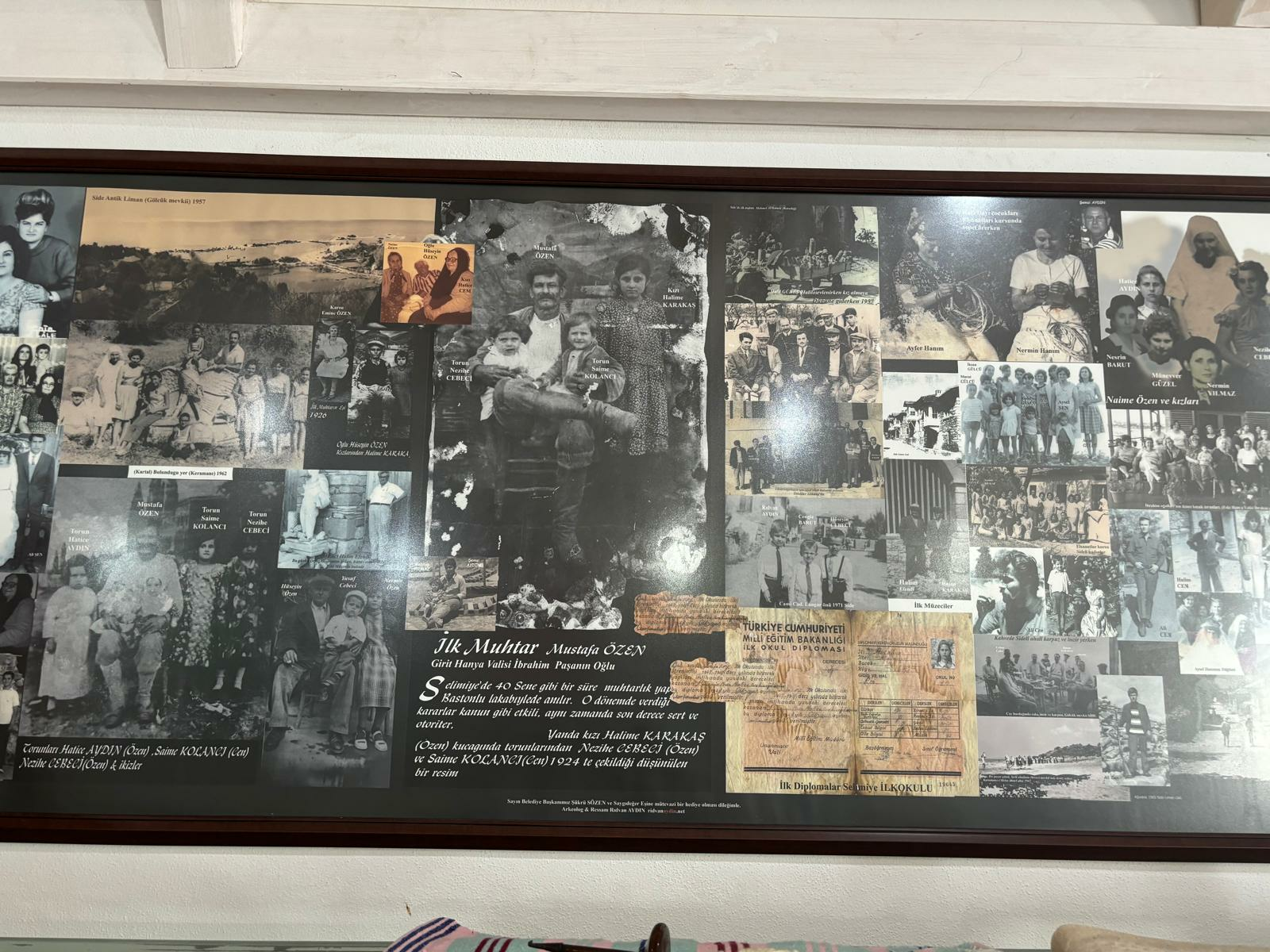

Supplement: S6 Fig — (TIF) [file pone.0314543.s006.tif]
